# 混合型固相萃取-高效液相色谱-串联质谱法测定稻米中3种谷维素

**DOI:** 10.3724/SP.J.1123.2021.12016

**Published:** 2022-08-08

**Authors:** Hongyan LI, Huan YANG, Chenyi MA, Wanyue ZHANG, Qingyu XU, Mingxue CHEN, Youning MA

**Affiliations:** 中国水稻研究所, 农业农村部稻米及制品质量监督检验测试中心, 浙江 杭州 310006; China National Rice Research Institute, Rice Quality Inspection and Supervision Center, Ministry of Agriculture and Rural Affairs, Hangzhou 310006, China

**Keywords:** 高效液相色谱-串联质谱, 混合型固相萃取, 谷维素, 稻米, high performance liquid chromatography-tandem mass spectrometry (HPLC-MS/MS), mixed-mode solid-phase extraction, oryzanol, rice

## Abstract

建立了利用高效液相色谱-串联质谱结合混合型阴离子交换固相萃取柱测定稻米中3种谷维素(环木菠萝烯醇阿魏酸酯(CA-FA)、24-亚甲基环木菠萝烯醇阿魏酸酯(24MCA-FA)和菜油甾醇阿魏酸酯(Camp-FA))含量的分析方法。实验通过优化3种谷维素的多反应监测(MRM)质谱参数,比较了在不同流动相中的分离度以及响应强度,同时考察了不同提取条件、净化条件对3种谷维素提取率和净化效果的影响,再结合外标法定量,实现了对稻米中3种谷维素的定量分析。实验结果表明,采用5 mmol/L乙酸铵水溶液为流动相A,甲醇∶乙腈=1∶1(v/v)为流动相B,梯度洗脱,3种谷维素在Agilent Eclipse XDB-C8色谱柱(150 mm×2.1 mm, 3.5 μm)上基本分离且响应强度和峰形最佳;提取条件经正交实验优化后可得:料液比为1∶20(g/mL)、提取溶剂为甲醇、浸泡时间为12 h、超声温度为40 ℃以及超声时间为20 min时,3种谷维素提取率最高;对混合型阴离子交换固相萃取柱的上样溶剂以及洗脱溶剂优化后,样品基质效应为1.6%~10.8%。在各优化条件下,3种谷维素在各自的线性范围内线性良好,相关系数(*r*^2^)均≥0.9983,检出限(LOD)为0.5~1.0 μg/L,定量限(LOQ)为2.0~3.5 μg/L。在稻米样品本底浓度2、5和10倍的加标水平下3种谷维素的平均回收率为86.1%~110.6%,相对标准偏差(RSD)为0.9%~3.2%。该方法可快速准确测定稻米中3种谷维素的含量,为后续稻米中谷维素类化合物测定及鉴定奠定基础。

## Abstract (en)

Rice is a major dietary staple in many communities owing to its high nutritional value and characteristic aroma. Oryzanol, a mixture of ferulic acid esters of triterpene alcohols and phytosterols, is a major group of phytochemicals found in rice. 24-Methylenecycloartanyl ferulate (24MCA-FA), cycloartenyl ferulate (CA-FA), and campestanyl ferulate (Camp-FA) have been identified as the primary components of oryzanol. At present, for the quantification of oryzanol in rice and rice products, UV spectroscopy or high performance liquid chromatography (HPLC) is widely employed. However, these methods cannot differentiate individual oryzanols, resulting in higher measured values. To extract oryzanol, methods including liquid-liquid extraction, acidulation extraction, and direct solvent extraction have been typically employed, as they do not require specific extraction instrumentation. However, there has been no systematic study on the direct solvent extraction and purification conditions of oryzanol in rice. In this study, a rapid and accurate analytical method based on HPLC-MS/MS and mixed-mode anion exchange (MAX) solid-phase extraction was established to determine the content of three oryzanols (24MCA-FA, CA-FA, and Camp-FA) in rice. The MS parameters, such as the collision energy of three ion pairs of each oryzanol, were optimized. Further, the chromatographic separation conditions and response intensities of the oryzanols in different mobile phases were compared. The effects of different pretreatment conditions on the extraction efficiency of the three oryzanols in rice samples and different purification conditions on their recovery were investigated. Combined with the external standard method, the three oryzanols in rice were successfully quantified. The results showed that the baseline separation and highest response for the three oryzanols were achieved using the Agilent Eclipse XDB-C8 chromatographic column (150 mm×2.1 mm, 3.5 μm) when methanol∶ acetonitrile in a 1∶1 ratio (v/v) and an aqueous solution of 5 mmol/L ammonium acetate were used as the mobile phases for gradient elution. The extraction rate of the three oryzanols was highest when using 2.5 g of the sample, adding 20 mL of methanol, soaking for 12 h, ultrasonicating at a temperature of 40 ℃ for 20 min, and centrifuging the extracted solutions at 4500 r/min for 10 min. The samples were purified by MAX, and the sample matrix effect was found to be lesser than 1.6%-10.8%. Under the optimum conditions, the calibration curves of the three oryzanols showed good linearity (correlation coefficients *r*^2^≥0.9983) within their respective linear ranges. The limits of detection were in the range of 0.5-1.0 μg/L, and limits of quantification were in the range of 2.0-3.5 μg/L. Accuracy and precision experiments were performed on rice samples spiked at three levels (2, 5, and 10 times the background concentration), with three replicates. The average recoveries of the three oryzanols ranged from 86.1% to 110.6%, and the relative standard deviations (RSDs) were between 0.9% and 3.2%. The method showed good performance when applied to the analysis of real samples. In conclusion, the developed method can determine the content of the three oryzanols in rice quickly and accurately, and can be used for the subsequent measurement of oryzanol compounds in rice.

稻米被誉为“天然营养素包”之一,富含人体所需的各种营养素及生理活性物质,如纤维素、矿物质、脂肪、谷维素和维生素E等^[1,2]^。谷维素大约在60年前首次在稻米中被发现,它是植物甾醇或三萜醇的阿魏酯混合物^[3,4]^,稻米中约85%的谷维素主要由3种化合物组成,分别是24-亚甲基环木菠萝烯醇阿魏酸酯(24MCA-FA)、环木菠萝烯醇阿魏酸酯(CA-FA)和菜油甾醇阿魏酸酯(Camp-FA)^[5]^。谷维素由于表现出较强的生物活性使得其在化妆品行业、营养和药物领域有着巨大的应用潜力^[6]^,如治疗高血压、消除脸部黑色素沉积、调节植物神经等作用^[7⇓-9]^。

现今谷维素类化合物的提取及测定主要集中在稻米米糠油方面的研究,而对稻米籽粒中谷维素类化合物的提取及测定的研究较少。因此,测定稻米中3种谷维素含量的方法研究,对于稻米制品以及稻米中谷维素类化合物的应用具有重要意义。

目前谷维素检测方法主要为紫外分光光度法^[10]^、高效液相色谱法(HPLC)和高效液相色谱-串联质谱法(HPLC-MS/MS)^[11]^等。其中紫外分光光度法操作简便,但由于样品中的杂质会在检测波长315 nm处出现较强吸收峰而导致测定结果偏高^[12]^; HPLC重复性高,但由于谷维素为混合物,HPLC很难测定单一的谷维素含量^[13]^,如王英瑛等^[14]^利用HPLC测定谷维素片中的谷维素时发现所有谷维素组分均在同一时间出峰,不利于单一谷维素的定量分析;HPLC-MS/MS利用液相色谱的分离功能和质谱的鉴定功能对样品进行分离、分析和鉴定,能精确测定谷维素含量,如Waraksa等^[15]^采用HPLC-MS/MS技术实现了营养补充剂中CA-FA、Camp-FA、24MCA-FA以及*β*-谷甾醇阿魏酸酯(*β*-Sit-FA)4种谷维素的含量测定。提取方法有溶剂萃取法^[16,17]^、分子印迹法^[18]^和超声辅助法^[19]^等。溶剂萃取法和分子印迹法操作时间较长,且溶剂萃取法所得谷维素纯度不高;超声辅助溶剂提取时可缩短实验时间并且有助于谷维素提取效率的提高,如王玉莹等^[20]^利用超声辅助乙醇提取米糠油中的谷维素时,发现谷维素的提取效率相对于传统溶剂提取法高14.31%左右。稻米样品基质复杂,含有淀粉、蛋白质、脂类、糖类等成分,对目标物容易造成干扰,因此上机前需要提取净化^[21]^。常见的净化方法有固相萃取、吸附法、液液萃取、膜富集法等。吸附法洗脱过程时间长,成品中也容易有溶剂残留;膜富集法由于目标化合物和基质中其他分子之间的大小差异太小,无法使用多孔膜实现进一步分离,所以只能实现目标化合物的初步富集^[16]^;固相萃取法应用最为广泛,其发展与吸附剂的性能密切相关,混合型固相萃取柱具有两种不同色谱分离基质(反相和离子交换),可以通过疏水性或离子作用实现选择性保留,从而获得更干净的提取物。

为此本研究对稻米中3种谷维素CA-FA、24MCA-FA和Camp-FA提取条件和质谱条件以及流动相种类等进行优化后,再利用混合型阴离子交换固相萃取柱净化样品溶液以消除杂质对目标物的干扰。综上,本文建立了适用于稻米中3种谷维素的检测方法,以期在实际样品的检测中得以应用,并为进一步研究稻米中谷维素的变化规律和鉴别提供技术手段。

## 1 实验部分

### 1.1 仪器与试剂

LC-20ADCR液相色谱仪(Shimadzu,日本); AB Sciex QTRAP 5500三重四极杆质谱仪(SCIEX,美国); KQ-300TED型高频数控超声仪(昆山市超声仪器有限公司);十万分之一天平(Mettler Toledo,美国); Milli-Q超纯水仪(Millipore,美国); MS3 basic漩涡振荡器、高速匀浆机(IKA,德国);氮吹仪(Organomation,美国); Primo R台式离心机(Thermo Fisher Scientific,德国);Oasis MAX固相萃取柱(Waters,美国)。

甲醇、乙腈、异丙醇(色谱级)购于德国Merck公司;氨水、甲酸、乙酸铵(色谱级)购于美国Sigma-Aldrich公司;标准品:24MCA-FA购于中国北京百威灵科技有限公司(纯度≥95%); CA-FA购于日本和光纯药株式会社(纯度≥99.4%); Camp-FA购于加拿大Toronto Research Chemical公司(纯度≥96%);实验室用水为Milli-Q超纯水。用于分析的稻米均由中国水稻研究所富阳基地种植所得。

### 1.2 标准溶液的配制

标准溶液的配制:用异丙醇配制Camp-FA质量浓度为100.0 mg/L, CA-FA和24MCA-FA质量浓度为1000.0 mg/L的标准储备液,于-20 ℃保存。分别吸取一定量的标准储备液,用甲醇配制成质量浓度1.0 mg/L的混合标准溶液,置于-20 ℃保存。

### 1.3 样品前处理

#### 1.3.1 超声提取

称取约2.5 g样品于100.0 mL离心管中加入50.0 mL甲醇,1000 r/min匀浆1 min,常温条件下浸泡12 h,静置完毕后在超声温度为40 ℃条件下超声20 min,以4500 r/min离心10 min,取10.0 mL上清液氮吹至近干,用30%甲醇水溶液(含1%氨水)定容至5.0 mL,待净化。

#### 1.3.2 固相萃取柱净化

将上述溶液转移至依次用5.0 mL甲醇和5.0 mL 1%氨水溶液活化后的MAX固相萃取柱,再分别用5.0 mL 1%氨水溶液和5.0 mL甲醇淋洗,最后用5.0 mL含4%甲酸的甲醇溶液洗脱,收集洗脱液,并用甲醇定容至10.0 mL,取上清液,过0.22 μm有机滤膜,滤液供HPLC-MS/MS测定。

### 1.4 色谱条件和质谱条件

#### 1.4.1 色谱条件

色谱柱为Agilent Eclipse XDB-C8(150 mm×2.1 mm, 3.5 μm),流动相A为5 mmol/L乙酸铵,流动相B为甲醇∶乙腈=1∶1(v/v);流速:0.35 mL/min;柱温:45 ℃;进样量:2.0 μL。梯度洗脱程序:0~3.0 min, 20%B~95%B; 3.0~14.0 min, 95%B; 14.0~14.1 min, 95%B~20%B; 14.1~20.0 min, 20%B。

#### 1.4.2 质谱条件

电离模式:电喷雾电离、负离子模式(ESI^-^);气帘气压力(CUR): 210 kPa;喷雾电压(IS): -4500 V;离子源温度(TEM): 450 ℃;雾化气压力(GS1): 310 kPa;辅助加热气压力(GS2): 240 kPa。多反应监测(MRM)模式采集,Analyst 1.6.3软件系统处理数据,相关质谱参数及离子信息见表1。

**表1:** 3种谷维素在多反应监测模式下的质谱参数

Compound	Parent ion (m/z)	Product ion (m/z)	Declustering potential/V	Collision energy/ eV
Campesteryl ferulate	575.4	560.4^*^	-50.45	-51.46
(Camp-FA)		193.0	-50.45	-49.25
Cycloartenyl ferulate	601.4	586.6^*^	-56.86	-55.60
(CA-FA)		175.0	-56.86	-55.89
24-Methylenecycloartenyl	615.5	600.5^*^	-63.45	-56.64
ferulate (24MCA-FA)		175.3	-63.45	-58.28

* Quantitative ion.

## 2 结果与讨论

### 2.1 色谱条件的优化

谷维素是弱极性化合物,且Agilent Eclipse XDB-C8色谱柱由硅胶键合辛烷基形成固定相,辛烷基极性大于十八烷基,且小于酰胺基团,因此更适用于分析弱极性化合物,故选择Agilent Eclipse XDB-C8色谱柱作为3种谷维素的分离柱。谷维素在甲醇、乙腈中均有良好的溶解性,所以选取甲醇和乙腈作为流动相B探讨3种谷维素分离度及色谱峰的变化。如图1a所示,当流动相B为乙腈时,色谱峰表现出较明显的劈叉、峰宽展宽以及不对称现象,且24MCA-FA色谱峰不明显;如图1b可知,当流动相B为甲醇时,色谱峰劈叉现象有所改善,峰形对称且尖锐,且3种谷维素均出现明显色谱峰。根据Camp-FA和CA-FA在乙腈中分离度为*R*_s_=1.05、Camp-FA和24MCA-FA在甲醇中的*R*_s_=1.18可知,单独选择一种溶剂作为流动相B不能有效地分离3种谷维素,因此尝试将甲醇和乙腈按一定比例混合。如图1c所示,当甲醇和乙腈以体积比1∶1混合作为流动相B时,3种谷维素相邻色谱峰之间的分离度分别为*R*_s1_=1.21、*R*_s2_=1.29,表明3种谷维素实现基本分离,故选择甲醇∶乙腈=1∶1(v/v)为流动相B。

**图1:**
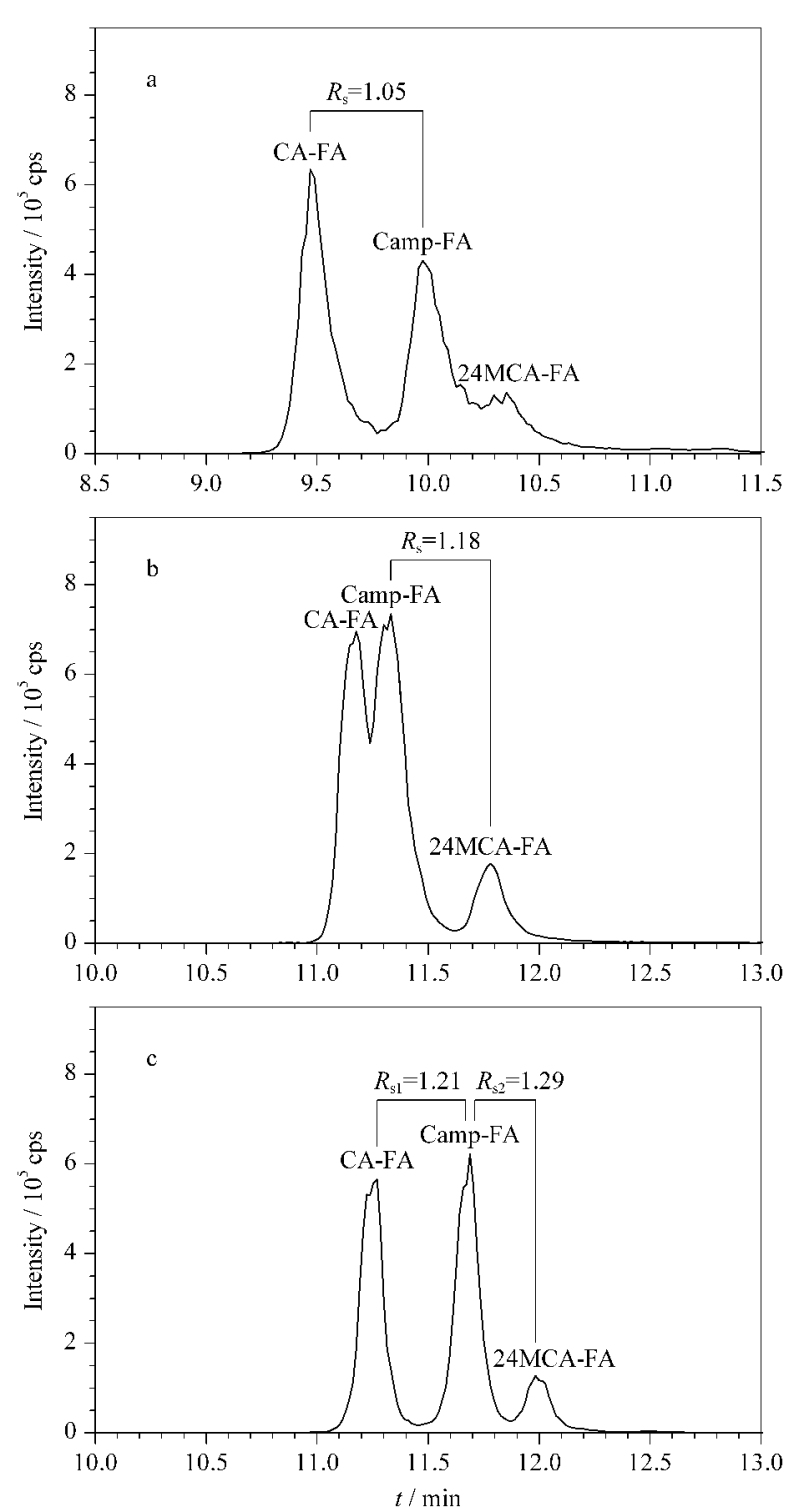
不同流动相B对3种谷维素分离度及色谱峰的影响

### 2.2 前处理条件的优化

#### 2.2.1 提取条件的优化

Kumar等^[22]^采用超声结合甲醇提取米糠中3种谷维素时,可获得较好的提取效果,但缺乏对提取效率影响因素的系统探讨。因此,设计5因素4水平L_16_(4^5^)的正交实验考察各因素对谷维素提取效率的影响。称取2.5 g样品,分别在不同料液比(即样品质量与提取液的体积比, g/mL)(1∶15、1∶20、1∶25、1∶30)、提取液(85%、90%、95%、100%甲醇)、浸泡时间(6、9、12、15 h)、超声温度(35、40、45、50 ℃)和超声时间(20、30、40、50 min)的条件下提取。

由表2极差*R*的大小可知,各因素对3种谷维素提取效率的影响依次为:提取液甲醇体积分数>浸泡时间>料液比>超声时间>超声温度。以3种谷维素提取效率作为参考指标,5个因素的最优提取组合为料液比1∶20 (g/mL)、甲醇体积分数100%、浸泡时间12 h、超声温度40 ℃以及超声时间20 min。

**表2:** 正交实验结果

No.	Solid-liquid ratio/ (g/mL)	φ(Methanol)/ %	Soak time/h	Ultrasonic temperature/ ℃	Ultrasonic time/min	Camp-FA/ (mg/kg)	24MCA-FA/ (mg/kg)	CA-FA/ (mg/kg)
1	1∶15	85	6	35	20	0.36	2.66	1.10
2	1∶15	90	9	40	30	0.42	3.46	1.42
3	1∶15	95	12	45	40	0.53	5.10	1.93
4	1∶15	100	15	50	50	0.52	3.45	1.31
5	1∶20	85	9	45	50	0.30	2.30	0.91
6	1∶20	90	6	50	40	0.58	4.56	1.80
7	1∶20	95	15	35	30	0.50	3.94	1.55
8	1∶20	100	12	40	20	0.63	6.16	2.24
9	1∶25	85	12	50	30	0.28	3.24	1.34
10	1∶25	90	15	45	20	0.38	3.82	1.56
11	1∶25	95	6	40	50	0.50	3.50	1.37
12	1∶25	100	9	35	40	0.50	3.22	1.25
13	1∶30	85	15	40	40	0.29	2.31	0.95
14	1∶30	90	12	35	50	0.47	3.68	1.47
15	1∶30	95	9	50	20	0.49	3.19	1.25
16	1∶30	100	6	45	30	0.56	4.30	1.63
K_1_	5.56	4.01	5.73	5.17	5.96			
K_2_	6.37	5.90	4.67	5.85	5.66			
K_3_	5.24	5.96	6.77	5.83	5.75			
K_4_	5.15	6.44	5.14	5.5	4.94			
R	1.22	2.43	2.09	0.65	1.02			

#### 2.2.2 净化条件的优化

MAX萃取柱适合分离纯化弱酸性化合物,鉴于谷维素弱酸、弱极性的特点,采用MAX萃取柱净化提取液。首先用5.0 mL甲醇和1%氨水活化MAX萃取柱,以30%、40%、50%、80%甲醇水溶液(含1%氨水)作为上样溶剂分别配制5.0 mL质量浓度为100 μg/L的混合标准溶液上样,通过3种谷维素的穿透率,考察甲醇体积分数对3种谷维素在MAX萃取柱穿透率的影响。结果如图2所示,当上样液为30%甲醇水溶液(含1%氨水)时,3种谷维素穿透率均<1%,随着甲醇体积分数的增加,穿透率逐渐升高,为确保3种谷维素能完全吸附于MAX萃取柱,确定上样溶剂为30%甲醇水溶液(含1%氨水)。

**图2:**
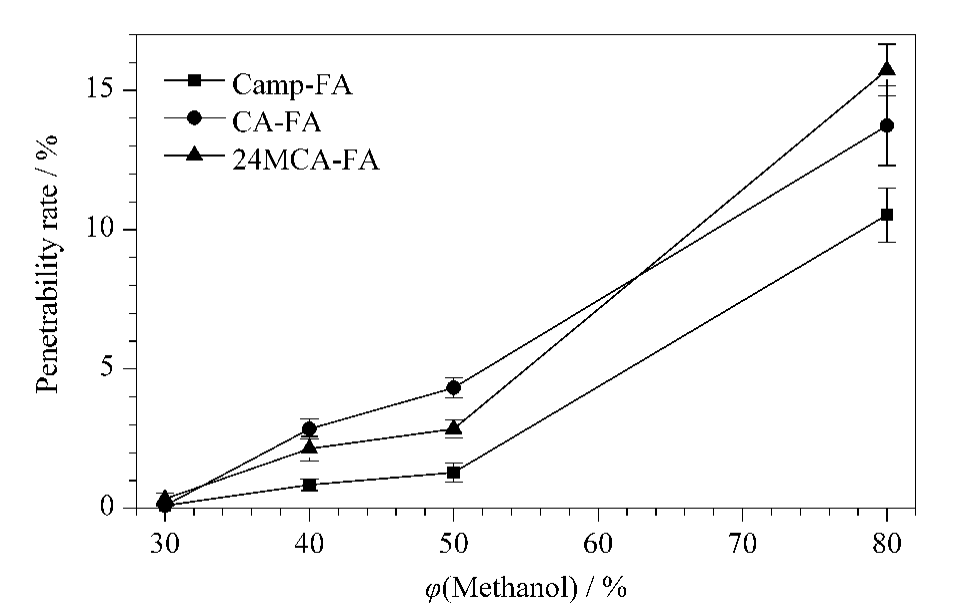
不同甲醇体积分数的上样溶剂对3种谷维素 穿透率的影响(*n*=3)

同时为保证3种谷维素能被完全洗脱,考察含1%、2%、4%、5%甲酸的甲醇洗脱液对回收率的影响。由图3结果表明,3种谷维素的回收率随着甲酸体积分数的增加表现为先升高后下降的趋势,当甲酸体积分数为4%时,3种谷维素几乎被完全洗脱,回收率为95.5%~100.4%。因此选用含4%甲酸的甲醇为洗脱液。

**图3:**
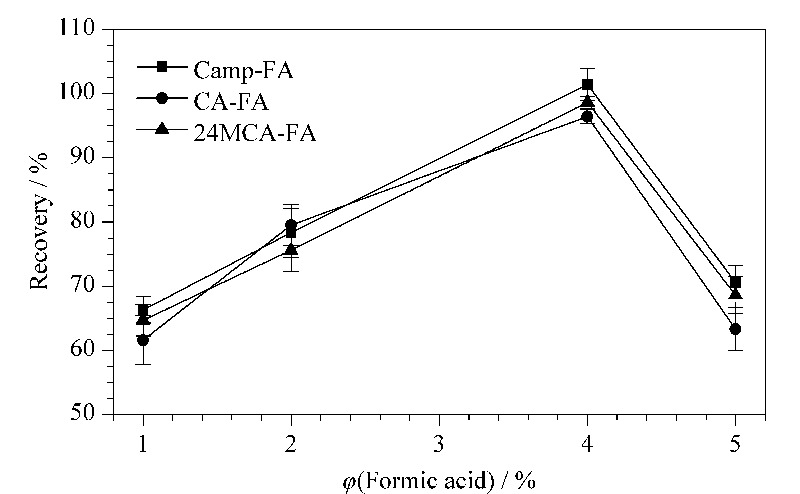
不同甲酸体积分数的洗脱液对3种谷维素 回收率的影响(*n*=3)

### 2.3 基质效应

基质效应是指在样品测定过程中,由于待测物以外其他物质的存在或其他物理、化学因素直接或间接影响离子化效果,从而影响待测物响应强度的现象^[23]^。本实验采用1.3节前处理方法分别制备未净化的样品溶液和净化后的样品溶液,分别以甲醇、未净化的样品溶液和净化后的样品溶液为溶剂,配制溶剂标准曲线和基质标准曲线,通过公式ME=(基质标准曲线的斜率/溶剂标准曲线的斜率-1)×100%计算3种谷维素的基质效应,以评价基质中杂质对分析物的影响^[24]^。由图4可知,未净化前CA-FA的ME为53.1%表现为基质增强效应,24MCA-FA的ME为-22.0%表现为基质抑制效应;净化后,3种谷维素ME为1.6%~10.8%,样品的基质效应减弱,表明样品基质对分析物的影响较小。

**图 4:**
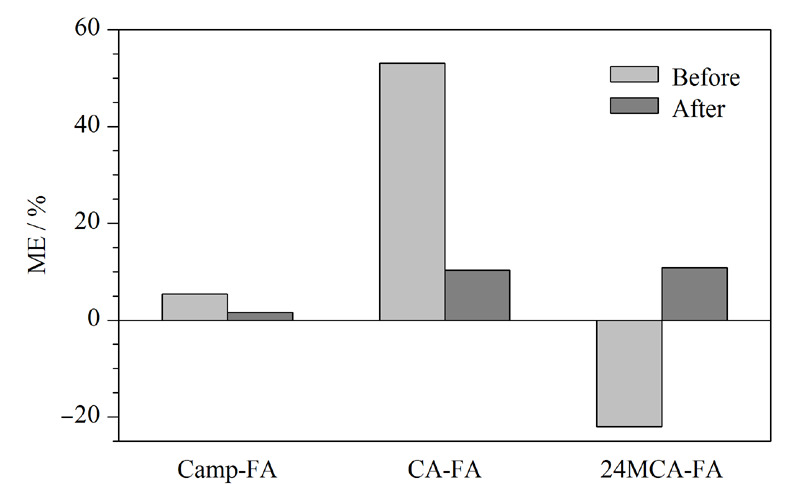
稻米中3种谷维素在净化前、后的基质效应

### 2.4 标准曲线、相关系数、检出限和定量限

将3种谷维素配制成系列浓度的标准工作液,以待测物峰面积为纵坐标,质量浓度(μg/L)为横坐标绘制标准曲线。结果如表3所示,Camp-FA、CA-FA在1.0~500.0 μg/L和24MCA-FA在5.0~1000.0 μg/L范围内呈良好的线性关系,相关系数(*r*^2^)均≥0.9983。以3种谷维素定量离子色谱峰的信噪比(*S/N*)为3和10计算检出限(LOD)和定量限(LOQ)。结果表明,3种谷维素的LOD为0.5~1.0 μg/L, LOQ为2.0~3.5 μg/L。

**表3:** 3种谷维素的线性方程、线性范围、相关系数、检出限和定量限

Compound	Linear equation	Linear range/(μg/L)	r^2^	LOD/(μg/L)	LOQ/(μg/L)
Camp-FA	y=2.45×10^5^x+1.21×10^4^	1.0-500.0	0.9991	0.5	2.0
CA-FA	y=2.31×10^5^x+6.33×10^3^	1.0-500.0	0.9992	0.5	2.0
24MCA-FA	y=4.24×10^4^x-9.44×10^3^	5.0-1000.0	0.9983	1.0	3.5

*y*: peak area; *x*: mass concentration, μg/L.

### 2.5 加标回收率和相对标准偏差

谷维素在稻米中本底含量相对较高,为进一步验证该方法测定稻米中3种谷维素的可行性,所以选择3种谷维素本底含量较少的稻米品种(扬粳722)进行加标回收试验。加标水平为稻米中3种谷维素本底含量的2、5和10倍,经1.3节步骤前处理,每个水平重复测定3次。由表4结果可知,3种谷维素的平均回收率为86.1%~110.6%,相对标准偏差为0.9%~3.2%,表明本方法具有良好的准确度和精密度。

**表4:** 稻米中3种谷维素的加标回收率及精密度(*n*=3)

Compound	Background/ (mg/kg)	Added/ (mg/kg)	Found/ (mg/kg)	Recovery/ %	RSD/ %
Camp-FA	0.3	0.6	0.96	110.1	2.6
		1.5	1.76	97.6	2.1
		3.0	3.17	95.5	1.0
CA-FA	0.5	1.0	1.58	108.5	0.9
		2.5	2.65	86.1	1.3
		5.0	5.27	95.5	2.1
24MCA-FA	2.5	5.0	8.03	110.6	1.6
		12.5	14.52	96.2	3.2
		25.0	28.40	103.6	3.1

### 2.6 在实际样品分析中的应用

为了验证本研究建立方法的适用性,实验最后采用建立的分析方法测定不同稻米中3种谷维素的含量,分析结果见表5。在7份稻米样品中均检测到3种谷维素,其含量为1.68~42.51 mg/kg。因此,该方法对稻米中谷维素类化合物含量的测定具有参考作用。

**表 5:** 实际样品中3种谷维素的含量测定结果

No.	Species	Sample name	Camp-FA/(mg/kg)	24MCA-FA/(mg/kg)	CA-FA/(mg/kg)
1	conventional Indica rice	Zhongzao 39	1.68	17.19	3.08
2		Huanghuazhan	2.92	25.57	5.50
3	conventional Japonica rice	Jia 67	2.06	17.19	9.28
4	Indica Hybrid rice	Zhongzheyou 8	5.57	42.51	8.95
5		Huazheyou 1	3.07	25.48	5.39
6	Japonica Hybrid rice	Jiayou 5	2.49	24.70	6.89
7	Indica Japonica Hybrid	Yongyou 1540	3.90	32.49	6.44

## 3 结论

本研究建立了高效液相色谱-串联质谱联用结合混合型阴离子交换固相萃取柱测定稻米中3种谷维素的分析方法。对前处理方法与色谱条件进行了优化,并进行了系列方法学验证。该方法具有操作简单、重复性强等特点,适合批量、快速检测稻米样品中3种谷维素的含量,并对谷维素类化合物的检测及应用具有一定参考价值。
